# Evaluation of Shear Horizontal Surface Acoustic Wave Biosensors Using “Layer Parameter” Obtained from Sensor Responses during Immunoreaction

**DOI:** 10.3390/s21144924

**Published:** 2021-07-20

**Authors:** Koji Kano, Hiromi Yatsuda, Jun Kondoh

**Affiliations:** 1 Japan Radio Co., Ltd., 834 Inasatomachi Nagano-shi, Nagono 381-2289, Japan; yatsuda.hiromi@tst.bio; 2 Graduate School of Science and Technology, Shizuoka University, Shizuoka 432-8561, Japan; kondoh.jun@shizuoka.ac.jp

**Keywords:** SH-SAW, biosensor, immunoreaction

## Abstract

Shear horizontal surface acoustic wave (SH-SAW) biosensors measure the reaction of capture antibodies immobilized on the sensing surface to capture test molecules (antigens) by using the change in SH-SAW propagation characteristics. SH-SAW displacement exists not only on the SH-SAW propagating surface, but also partially penetrates the specimen liquid to a certain depth, which is determined by the liquid properties of the specimen and the operating frequency of the SH-SAW. This phenomenon is called viscosity penetration. In previous studies, the effect of viscosity penetration was not considered in the measurement of SH-SAW biosensors, and the mass or viscosity change caused by the specific binding of capture antibodies to the target antigen was mainly used for the measurement. However, by considering the effect of viscosity penetration, it was found that the antigen–antibody reaction could be measured and the detection characteristics of the biosensor could be improved. Therefore, this study aims to evaluate the detection properties of SH-SAW biosensors in the surface height direction by investigating the relationship between molecular dimensions and SH-SAW propagation characteristics, which are pseudo-changed by varying the diameter of gold nanoparticles. For the evaluation, we introduced a layer parameter defined by the ratio of the SH-SAW amplitude change to the SH-SAW velocity change caused by the antigen–antibody reaction. We found a correlation between the layer parameter and pseudo-varied molecular dimensions. The results suggest that SH-SAW does not only measure the mass and viscosity but can also measure the size of the molecule to be detected. This shows that SH-SAW biosensors can be used for advanced functionality.

## 1. Introduction

The global outbreak of COVID-19 in 2020 has reminded everyone of the enormous impact of infectious diseases on industrial activities, social life, and people’s lives. Effective measures to prevent the spread of COVID-19 include testing and isolation. Two types of tests exist: antigen tests to detect the virus itself and tests to detect antibodies that neutralized the virus if it entered the body. The difference is that the former test detects the infection at the time of testing, while the latter test detects the presence of antibodies in the body that developed as a result of past infection with the virus or vaccination. Despite this difference, the requirements for these tests are common, that is, simplicity of the test procedure, low workload for medical personnel, rapid results, and capability to perform the test on-site. On-site diagnostic tests that require field-portable medical devices are categorized under point-of-care testing (POCT). Shear-horizontal surface acoustic wave (SH-SAW) technology-based [1,2,3,4,5,6] biosensors are considered promising candidates for the fundamental technology of diagnostic POCT devices because of their high portability and operability owing to their high affinity for mobile devices [7].

The SH-SAW biosensor measures the concentration of target specimens, commonly referred to as antigens in the context of antigen–antibody reactions, by measuring the amount of antigen bound onto surface-immobilized antibodies, which can bind targeted antigens [8]. Healthcare workers can analyze the concentration of testing antigens by measuring the velocity and amplitude of the SH-SAW characteristics. The rate of velocity and amplitude change, which can be rephrased as sensitivity, increases with the square root of the increasing SH-SAW biosensor operating frequency. There have been many attempts to improve the sensitivity of SAW sensors by increasing their operating frequency [9,10]. Apart from this approach, that is, increasing the operating frequency, a method of adding another substance to the target specimen as a means of increasing sensitivity is commonly used [11]. In our recent studies, we found that the properties of SH-SAW are affected not only by the mass of the measurement object but also by its dimensions [12]. These properties reflect the relationship between the dimensions of the measurement target specimens and the phenomenon in which SH-SAW penetrates surface-loaded specimens [13]. The penetration depth is determined by the operating frequency of the SH-SAW, which can be rephrased as the wavelength and viscosity of the specimen solvent. These findings suggest that the selection of the optimum mass, dimensions, and wavelength of the SH-SAW is necessary to maximize the performance of the SH-SAW biosensor. This study aims to clarify the behavior of the SH-SAW biosensor in the height direction by measuring various specimens with specific dimensions. To this end, we explored the optimum parameters between an antigen, an antibody, and the wavelength of the changing particle size of the SH-SAW, which is attached to the secondary antibodies.

## 2. Materials and Methods

The correlation between the SH-SAW and antigen–antibody reaction occurring in the sensing area of the SH-SAW sensors was investigated to understand the behavior of the SH-SAW biosensor in the height direction. In a series of evaluations, C-reactive protein (CRP) [14] was measured for the SH-SAW biosensor. CRP is a protein whose concentration increases in the serum when inflammation or tissue destruction occurs within an organism. As a physical characteristic, the CRP molecule is a pentamer comprising five identical subunits. CRP molecules are approximately 11 nm in diameter and 118 kDa in weight [15]. A sandwich assay was used for the antigen–antibody reaction. The sandwich assay in a surface acoustic wave device is performed by adding secondary antibodies for signal amplification to the detected antigens that have reacted with the capture antibodies immobilized onto the sensor surface. Both capture and secondary antibodies employed immunoglobulin G (IgG)-classified monoclonal antibodies. The molecular size of IgG is 10–15 nm [16,17] in height and 150 kDa in weight [18]. The variation in the biomolecular size was prepared by changing the gold nanoparticle [19,20,21,22] diameters and gold nanoparticles conjugated with secondary antibodies. The measured diameters of the gold nanoparticles were 10, 15, 20, and 30 nm. The SH-SAW biosensor devices used in the series of evaluations were operated at 250 MHz. The experimental conditions are presented in Table 1.

### 2.1. SH-SAW Biosensor Device

**Figure 1:**
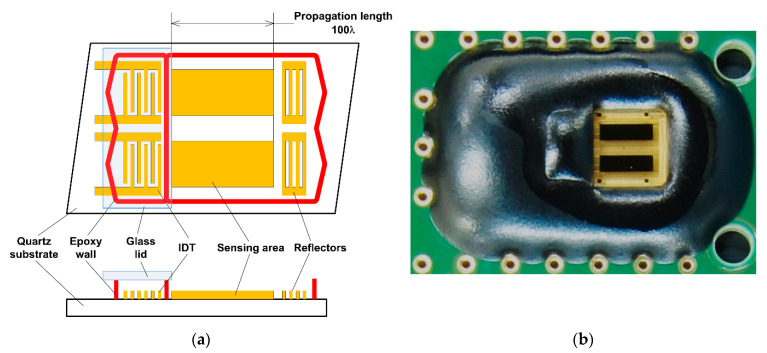
SH-SAW biosensor device used in this study. (**a**) Schematic of the top view and cross section; (**b**) picture of the sensor device. Only the sensing areas and reflectors are exposed; the other parts are covered with black epoxy resin.

To evaluate the sensing characteristics of the SH-SAW biosensor for different molecular dimensions, we fabricated and used a 250 MHz SH-SAW sensor device. This device was fabricated on a 36Y-90X quartz substrate and has a reflective structure comprising interdigital transducers, IDTs, a sensing area, and reflectors. These components were manufactured using a deposited 92 nm gold thin film. The IDTs convert the input electrical signals applied by the measuring circuit to the sensor device into SH-SAWs, which are then supplied to the sensing area. IDTs also convert the SH-SAW reflected by reflectors received from the sensing area back into electrical signals, which are supplied to the measuring circuit. The IDTs are surrounded by an epoxy wall structure and are covered with a glass lid to prevent short-circuiting due to contact with the evaluating specimen liquids. The sensing area has a length of 2000 μm and a 4000 μm round trip, which correspond to 100 and 200 wavelengths, respectively. The sensing area located between the IDTs and reflectors is covered with capture antibodies that detect the targeted substance; such materials are generally called antigens. The electrical signals applied to the sensor device from the measuring circuit were electromechanically converted to an SH-SAW. The SH-SAW propagated across the sensing area toward the reflectors. After reaching the reflectors, the SH-SAW was reflected, passed through the sensing area again, and then returned to the IDTs. The SH-SAW was converted into an electric signal and re-emitted into the measuring circuit. While propagating through the sensing area, the SH-SAW propagating characteristics were modified by the nature and conditions of molecules and liquid surfaces loaded on the sensing area. The propagating characteristic modifications appear in the form of velocity and/or amplitude changes in the SH-SAW. The propagating characteristic modifications reflect the change in shape, that is, the size of the surface molecules caused by the capture of its target antigen by the surface-immobilized antibody. The schematic and photograph of the fabricated device are shown in Figure 1, and its design parameters are listed in Table 2.

### 2.2. Measurement Electronics

**Figure 2:**
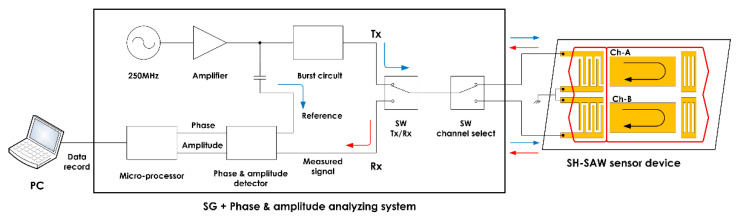
Block diagram of the measurement system.

A measurement system to detect the phase and amplitude was developed to analyze the antigen–antibody reaction using the modified SH-SAW propagating characteristics. The developed system comprised a 250 MHz oscillator operating in burst mode, a circuit for detecting the phase and amplitude differences between the input and output signals, and radio frequency (RF) switches for signal control. By using burst signals, the SH-SAW response and electromagnetic responses of the sensor device can be separated. Owing to the separation of the electromagnetic response, the propagation characteristics of SH-SAW modified by antigen–antibody reactions can be measured precisely. The detecting circuit compares the transmitted signal with the returned signal of the sensor device to measure the phase and amplitude changes of the sensor device. RF switches located between the burst oscillator, detection circuit, and sensor device control the transmission and reception of the measurement signals. In addition, the RF switches can select one of the multiple measurement channels integrated inside the sensor device, realizing multiple independent measurements with a single sensor device. A block diagram of the measurement system is shown in Figure 2. We integrated this measurement system into an integrated circuit (IC) to realize a compact and low-cost system with high performance.

### 2.3. Surface Structures: Antigen, Antibody, and Gold Nanoparticle

**Figure 3:**
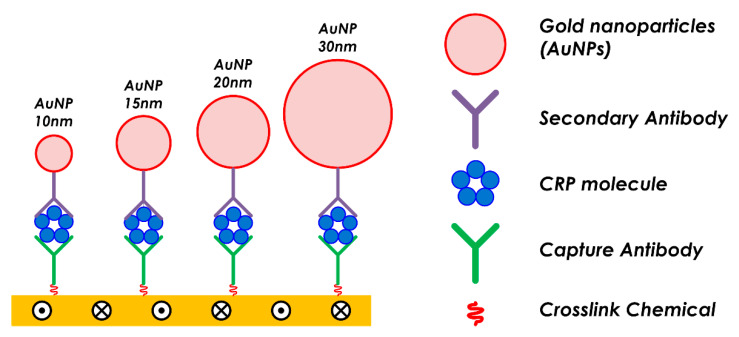
Schematic of the molecule structure with different gold nanoparticle diameters.

A sandwich assay to detect C-reactive protein (CRP) was employed to evaluate SH-SAW sensor measurement characteristics in specimens with different molecular sizes. The sandwich assay was performed by reacting with targeting proteins. In the sandwich assay, we used CRP with capture antibodies. Subsequently, secondary antibodies were used for amplifying the sensor output signals reacted with the CRPs captured in the previous step. The capture antibodies were immobilized onto the sensing area of the SH-SAW biosensor using a crosslinking chemical (dithiobis[succinimidylpropionate]), DSP, #22585, Thermo Scientific, Waltham, MA, USA). The HyTest (Turku, Finland) 4C28-CRP30 monoclonal antibody was used as the capture antibody. We used recombinant CRP (CRP Calibrator L-710, SHINO-TEST CORP., Tokyo, Japan) in a series of measurements. The HyTest 4C28-CRP135 monoclonal antibody was used as the secondary antibody. The secondary antibodies were conjugated with gold nanoparticles with diameters of D = 10, 15, 20, and 30 nm (EMGC10, EMGC15, EMGC20, and EMGC30, BBI Solutions, Crumlin, UK) to vary the molecular size. A structural diagram of the antigen, antibody, and gold nanoparticles on the SH-SAW biosensor surface is shown in Figure 3.

### 2.4. Gold Nanoparticle Conjugation Procedures

**Figure 4:**
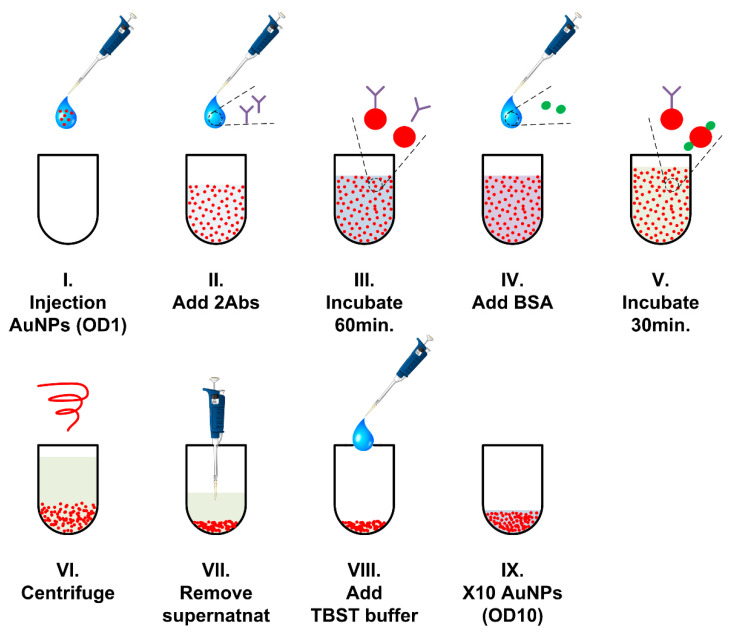
Process of conjugating secondary antibodies and gold nanoparticles.

Gold nanoparticles and anti-CRP antibody complexes were combined using electrostatic interactions to prepare molecules of various sizes [23]. The process of conjugating the secondary antibody to AuNPs is shown in Figure 4. The fundamental concentration of the gold nanoparticle dispersed solution, optical density 1 (OD-1), was injected into an empty tube (I). Secondary antibodies diluted in TES buffer were added to the gold nanoparticle solution to regulate the 1/10 concentration of the final concentration (II). Gold nanoparticles with secondary AuNPs were incubated for 60 min in a temperature-controlled clean room environment at 23 ± 1 °C (III). After incubation, 2% bovine serum albumin (BSA) was added to block the unreacted surface of gold nanoparticles (IV). The solution was incubated for 30 min to block the unreacted surface (V). Centrifugal operation was performed to separate the secondary antibody-conjugated gold nanoparticles from the buffer solution (VI). The separated buffer solution and supernatant were removed using a pipette (VII). After removing the supernatant with a pipette, TBST buffer (VIII) was added. The gold nanoparticle solution was concentrated 10-fold, OD-10, by reducing TBST to 1/10 the volume of initial gold nanoparticle liquid volume (IX). The stability of the gold nanoparticle-antibody complex is shown in the Supplementary Material.

### 2.5. Measurement Protocol

**Figure 5:**
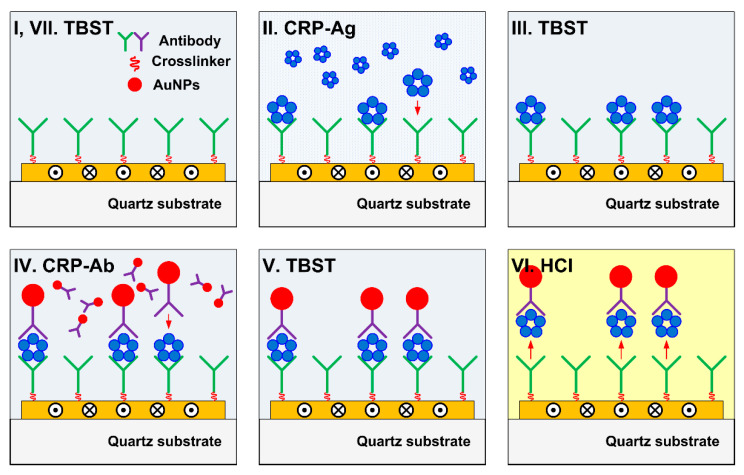
Surface condition in each measurement step.

The sensing characteristics of SH-SAW were evaluated by determining the antigen–antibody reaction to the sensing area of the SH-SAW biosensor device. In the evaluation process, recombinant CRP and gold nanoparticle-conjugated secondary antibodies were applied to the sensing area and the propagation characteristics of the SH-SAW were measured. The recombinant CRP and gold nanoparticle-conjugated secondary antibodies were measured consecutively; however, each step was evaluated separately. Figure 5 shows the surface conditions of the sensing area and the reacting molecules in each measurement step, and Table 3 summarizes the measurement procedure.

Before applying specimen molecules onto the sensing area, a reference solution, Tris-buffered saline with Tween (TBST), was used in the series of measurements. The solution was loaded onto the sensing surface to set the measurement standard (measurement step I). The sensor device was left for 60 s until there was a sufficiently reduced signal disturbance caused by the TBST load onto the sensing area. The TBST standard buffer was then replaced with the recombinant CRPs to measure the SH-SAW response toward antigen binding onto the surface-immobilized capture antibodies. One of the four CRP concentrations (0, 6, 16.5, and 30 μg/mL) was measured by selecting one for each cycle. The CRP antigens were titrated at a 10-fold dilution in phosphate-buffered saline (PBS) buffer to ensure the dynamic range of the measurements.

In a series of measurements, the incubation time for the antigen–antibody reaction was set at 180 s (measurement step II). This incubation time was determined by considering the saturation of the reaction; however, the volatilization of the specimen liquids did not affect the measurements. After the capture antibodies reacted with CRP molecules, the solution in the sensing area was replaced with TBST standard buffer to remove the remaining unreacted suspended molecules (measurement step III). As in measurement step I, step III required 60 s to remove the response disturbance caused by the liquid manipulation. The TBST standard buffer was then replaced with specimen solution to measure the SH-SAW response to secondary antibody binding onto the CRP molecule captured sites (measurement step IV). Similar to the CRP reaction in step III, the antigen–antibody reaction time was set to 180 s.

We reapplied the standard TBST to replace the unreacted gold nanoparticle-conjugated secondary antibodies (measurement step V). With the measurements in steps I–V, the evaluation was nearly completed. In this series of evaluations, the antigen–secondary antibody complexes were repeatedly released from surface-immobilized capture antibodies using the same sensor device (measurement step VI). To capture the antibody regeneration, a 100 mM concentration of HCl was used. When the pH value around the capture antibody changed (TBST: pH 7.5, → 100 mM HCl, pH 1.0), the shape of the antibody molecule changed. Some capture antibodies are known have degraded affinity when exposed to HCl. Therefore, in this series of evaluations, we selected the capture antibody, HyTest CRP-C30, that does not degrade even after repeated regeneration with HCl. As a result, the capture antibody releases bound CRP molecules and secondary antibody complexes. The durability of capture antibody to HCl regeneration process is shown in the Supplementary Material.

Finally, the 100 mM HCl step with the standard buffer TBST (measurement step VII) was repeated. As this surface condition is the same as that of measurement step I, the next cycle measurement was started.

**Figure 6:**
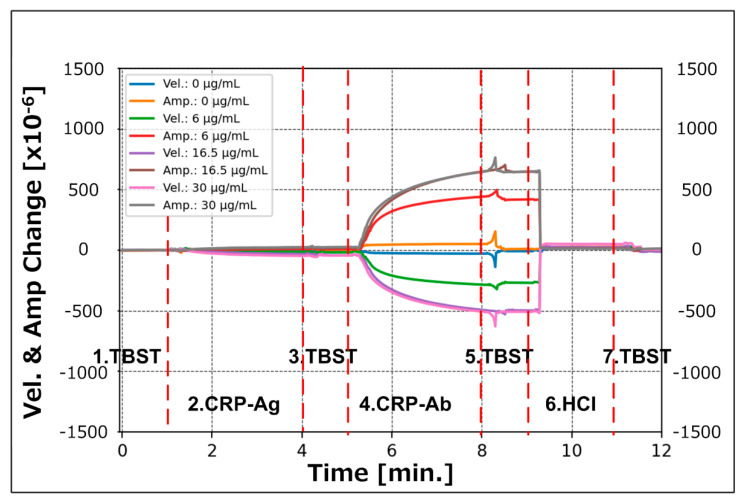
Recorded velocity and amplitude change in a typical measurement cycle.

Figure 6 shows the typical velocity and amplitude changes during the measurement cycle. In this figure, the horizontal axis shows the elapsed time from the start of measurement step I. The vertical axis shows the velocity and amplitude changes of the SH-SAW simultaneously. The negative region of the graph indicates a decrease in the SH-SAW velocity in the molecular capture, and the positive region indicates an amplitude change. As the amplitude change is defined as positive when the amplitude decreases, the positive change in the graph represents the attenuation of the SH-SAW.

A series of measurements were performed in a clean room environment with a controlled temperature of 23 ± 1 °C. As the measurements were performed using a quartz-based device with excellent temperature stability in a temperature-controlled environment, and no adverse effects of temperature fluctuations were observed in the measured results.

## 3. Results

### 3.1. Measured Time Course Data

**Figure 7:**
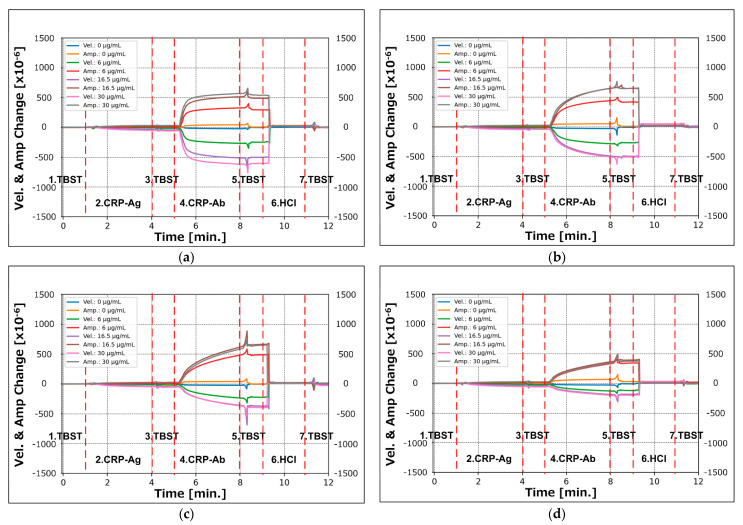
Recorded time-course response of velocity and amplitude changes in the SH-SAW for the CRP assay process. Secondary antibodies conjugated with gold nanoparticles of diameters of (**a**) 10 nm; (**b**) 15 nm; (**c**) 20 nm; (**d**) 30 nm.

The four graphs in Figure 7a–d illustrate the time responses of the measured velocity and amplitude changes for gold nanoparticle diameters of 10, 15, 20, and 30 nm; the graphs depict the measured data at one-second intervals for all measurement steps with the CRP concentrations of 0, 6, 16.5, and 30 μg/mL. The horizontal axis of each graph shows the elapsed time in minutes. In this series of evaluations, we performed four measurements for each condition; the graphs in Figure 7 show one datapoint extracted from all acquired data.

### 3.2. A/V Plane Plot

**Figure 8:**
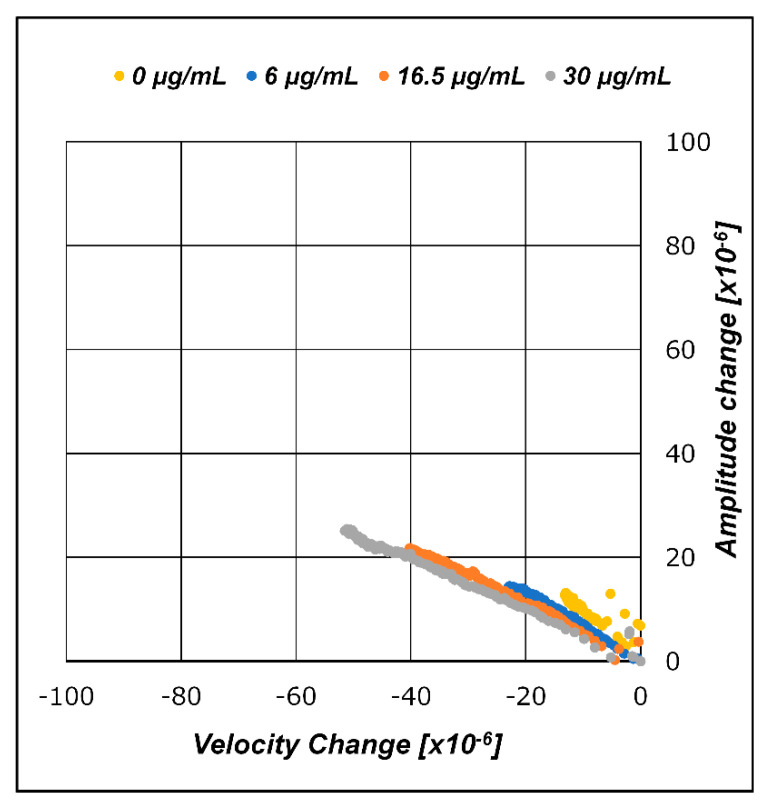
A/V plane plot for CRP molecule-binding step.

**Figure 9:**
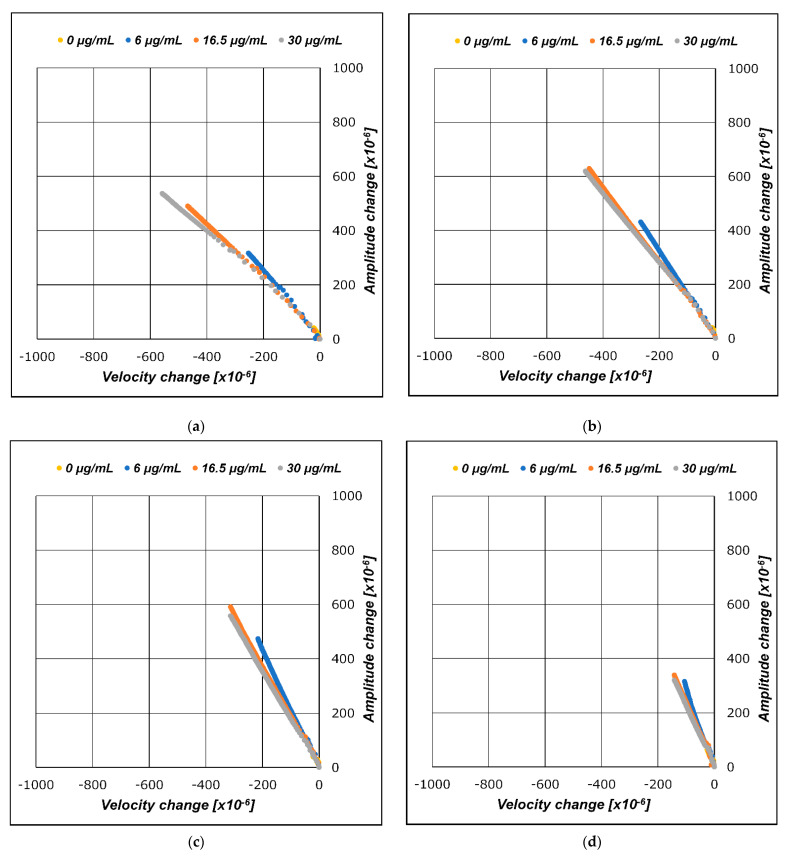
A/V plane plot for gold nanoparticle-conjugated secondary antibody-binding step. Secondary antibodies conjugated with gold nanoparticles of diameters of (**a**) 10 nm; (**b**) 15 nm; (**c**) 20 nm; (**d**) 30 nm.

As described in Section 3.1, we plotted the measured data on an A/V plane, which is a Cartesian plane that plots the velocity changes of the SH-SAW on the horizontal axis and the amplitude changes on the vertical axis. The velocity and amplitude changes of the SH-SAW in the CRP molecule adsorption process with regard to the capture antibodies were plotted in the A/V plane, as shown in Figure 8. The absolute values of the velocity and amplitude changes in the adsorption process were smaller than those of the gold nanoparticle-conjugated secondary antibody process in the later stages. This is reflected in the dimensions of the CRP molecule. The graphs of the velocity and amplitude changes plotted onto the A/V plane with respect to the gold nanoparticles adsorbed on the captured CRP antibodies are illustrated in Figure 9. The measured data obtained for different gold nanoparticle diameters (10, 15, 20, and 30 nm) are plotted in Figure 9a–d.

## 4. Discussion

### 4.1. Introduction to Layer Parameter and Evaluation of Antigen–Antibody Reaction

**Figure 10:**
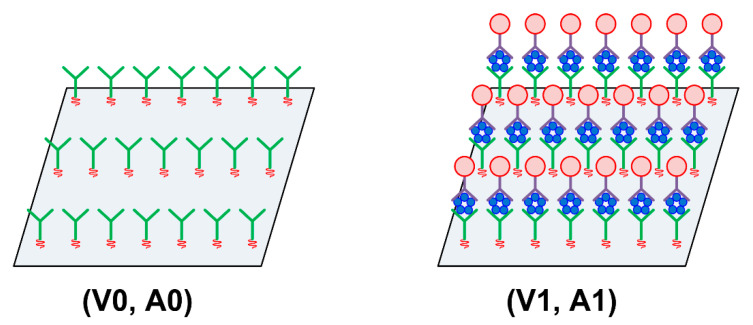
Surface images comparison of completely unreacted and reacted sensing areas.

To quantitatively discuss the experimental results described in Section 3.2, the ratios of amplitude and velocity changes, A/V, were calculated and evaluated. The changes in SH-SAW velocity and amplitude increase with the number of events in the antigen–antibody reaction, wherein the CRP antigens are bound to capture antibodies or the secondary antibodies are bound to the CRP antigen. In Figure 10, the fully antigen–antibody reacted and the unreacted states of the capture antibody-immobilized sensing area of S0 are compared. The SH-SAW velocities and amplitudes propagating on the fully reacted surface are described as V1 and A1, respectively, and those propagating on the unreacted surface are denoted as V0 and A0, respectively.

**Figure 11:**
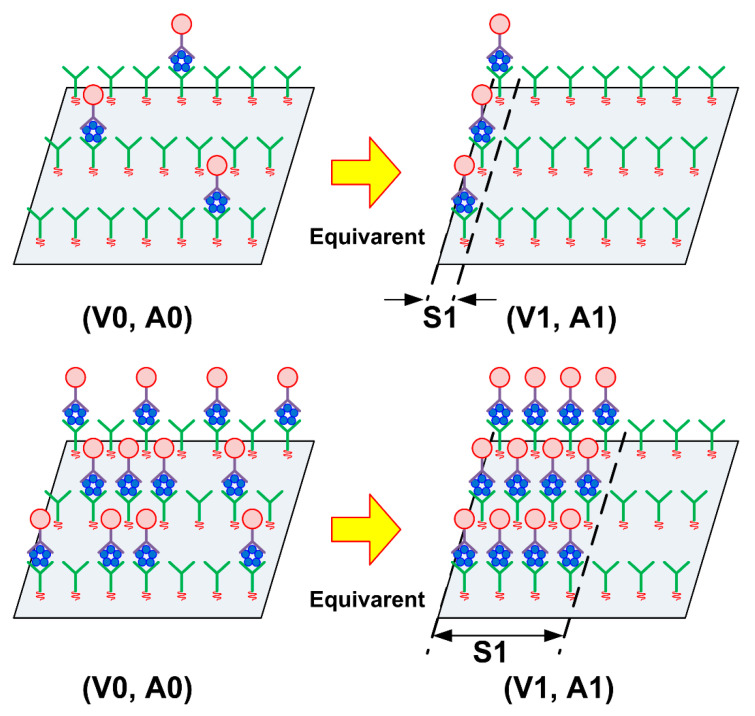
Comparison of number of antigen–antibody reactions on the sensing area.

Figure 11 shows a comparison of the numbers of antigen–antibody reactions. The upper part of Figure 11 shows fewer antigen–antibody reactions, and the lower part shows a case with several reactions. The left half of Figure 11 shows a situation where antigen–antibody reactions occur randomly in the sensing region, similar to the reactions on the actual device, and the right half of Figure 11 shows the classification of reacted (S1) and unreacted sites (S0–S1). The reacted site (S1) was considered proportional to the concentration of CRP antigen. The SH-SAW velocity and amplitude changes can be explained by the difference in the SH-SAW velocity and amplitude between the reacted surface of the antigen antibody and the unreacted surface. Using the ratio of the total area of the sites where the antigen–antibody complex formed to the total area of unreacted sites, velocity change (ΔV) and amplitude change (ΔA) are described using the following equations.

ΔV = (V0 − V1) × (S1/S0), (1)

ΔA = (A0 − A1) × (S1/S0),(2)

Using Equations (1) and (2), the ratio of the SH-SAW amplitude changes and velocity changes, ΔA/ΔV, can be calculated, as shown in Equation (3).

ΔA/ΔV = (A0 − A1)/(V0 − V1),(3)

The disappearance of the antigen concentration-dependent term (S1/S0) from Equation (3) suggests that the ΔA/ΔV ratio is a unique value representing a measurement target’s properties other than concentration, such as shape, viscoelastic coefficient, and density. It is assumed that the ΔA/ΔV ratio reflects the properties or structure of the biomolecules stacked on the SH-SAW biosensor surface; we called it the “layer parameter,” which is defined as the ratio of SH-SAW amplitude changes to velocity changes in the antigen–antibody reaction.

### 4.2. Correlation between Traces on Layer Parameter (ΔA/ΔV) and Diameters of Gold Nanoparticles

The layer parameter is a value defined by the ratio of amplitude change to velocity change, ΔA/ΔV, and is equivalent to the slope of the regression line of the measured data plotted on the A/V plane. The layer parameter values correspond to the line-plotted slopes on the A/V plane shown in Figure 8 and Figure 9a–d.

The graphs in Figure 8 and Figure 9a–d show the merged results of the entire measurement results for the evaluated CRP concentrations. Although Figure 8 and Figure 9a–d may look similar, they have slightly different implications. In the reaction where CRP is captured by the capture antibodies immobilized onto the SH-SAW biosensor sensing area, CRPs can be absorbed in all capture antibody sites.

Conversely, in gold nanoparticles, conjugated secondary antibody complexes are absorbed only in the sites where the surface capture antibodies bind the CRPs. In other words, the maximum number of reactions of the secondary antibody and gold nanoparticle complexes is restricted by the amount of CRP bound in the antigen-binding step. This phenomenon occurs because the total quantity of the secondary antibodies and gold nanoparticle complexes to be dropped is in excess compared to the amount of surface CRP trapped in the site. Thus, although the transients have different implications, the results are expected to be practically the same if the reaction time is set to be sufficiently long until the reaction is saturated. From Figure 8 and Figure 9a–d, it can be seen that the layer parameter is preserved in the applied CRP concentration change unless the molecular structure is unchanged. The CRP concentration changes and the layer parameter is kept constant. As the concentration changes, the length of the line, which is the set of amplitude and velocity measurements of the SH-SAW, changes with a constant slope.

**Figure 12:**
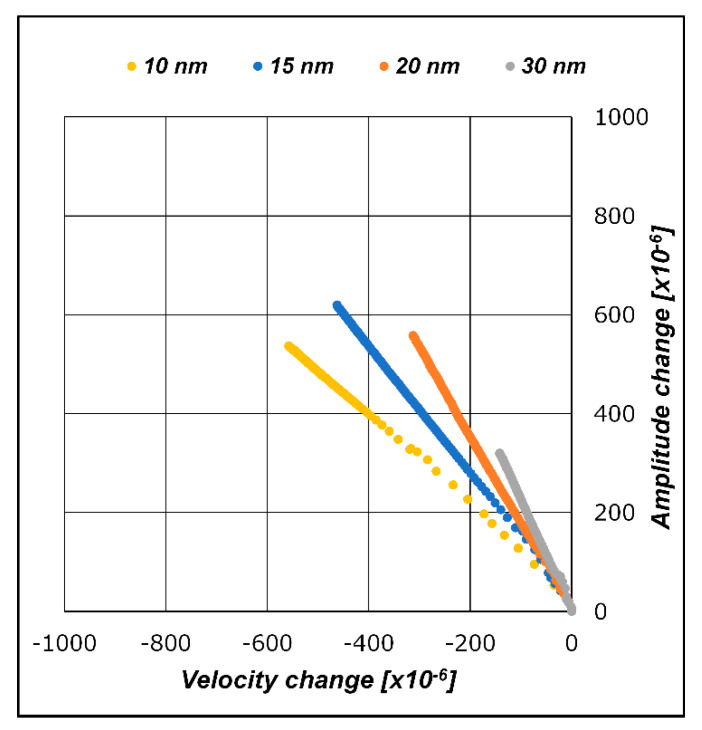
Layer parameter change with gold nanoparticle diameters (CRP concentration: 30 μg/mL).

The relationship between the diameter of the gold nanoparticles conjugated to the secondary antibodies and the layer parameters was investigated. Figure 12 shows the evolution of the SH-SAW velocity change trajectory and amplitude changes on the A/V plane when the conjugated gold nanoparticle diameter changes. Table 4 lists the layer parameter values as the regression line slopes of the measured results in each graph. Figure 3 illustrates the structure of the gold nanoparticles by particle diameter and antigen/antibody molecules formed on the sensing area after the measurements. Figure 12 and Table 4 show a significant increase in the amplitude change with increasing gold nanoparticle diameter. Accordingly, the absolute value of the layer parameter increases, that is, the slope of the lines on the A/V plane becomes steeper. This relationship also applies to the CRP measurement step, where no gold nanoparticles are attached to the molecules.

In the measurements with varying CRP concentrations, the SH-SAW velocity and amplitude changes were plotted on a particular line in the A/V plane and did not depend on the population of molecules in the sensing area but only on the dimensions of the molecules to be measured. These results suggest that the layer parameter, which is calculated from the SH-SAW velocity and amplitude changes, changes to reflect the dimensions and shape of biomolecules applied to the SH-SAW biosensor.

### 4.3. Verification of Measured Data Using Simulation

**Figure 13:**
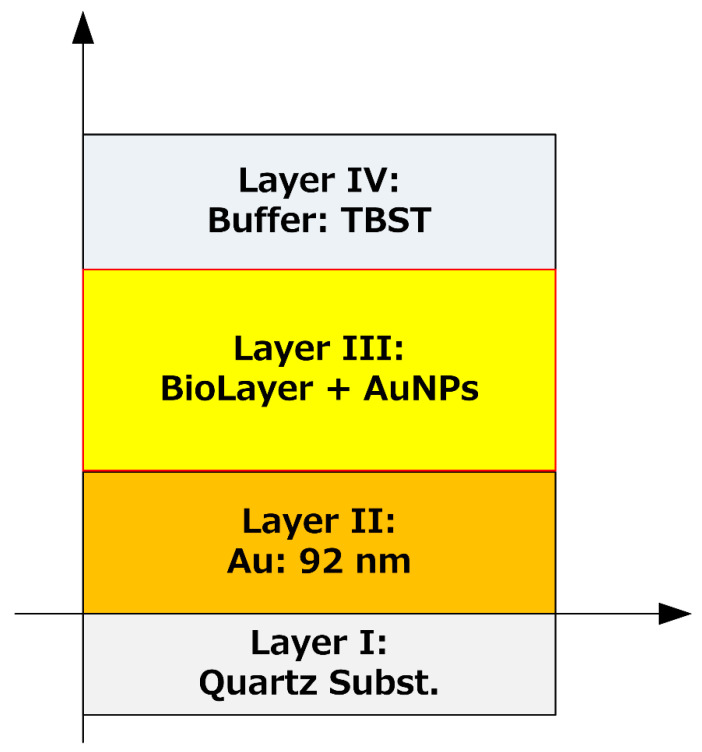
Calculated biosensor and biolayer structures.

The measurement characteristics of SH-SAW biosensors were determined by the height dimension of the molecules formed on the sensing surface, as suggested by the experimental results; they were also verified through numerical simulations. A modified simulation was used as a numerical simulation method by adding viscosity effects to the Campbell and Jones method [24,25,26]. The numerical simulation assumes a four-layer structure: SAW substrate (quartz), sensing electrode (gold thin film, thickness: 92 nm), biomolecule layer (capture antibody, CRP, secondary antibody, and gold nanoparticle complex), and buffer solution layer. A schematic of the calculation model is presented in Figure 13. The simulation calculates the SH-SAW velocity and amplitude as the thickness of the biomolecule layer changes.

**Figure 14:**
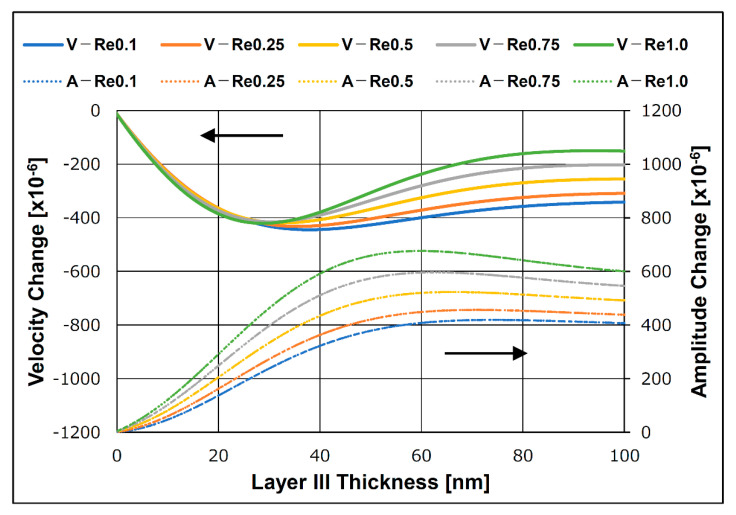
Calculated velocity and amplitude changes.

**Figure 15:**
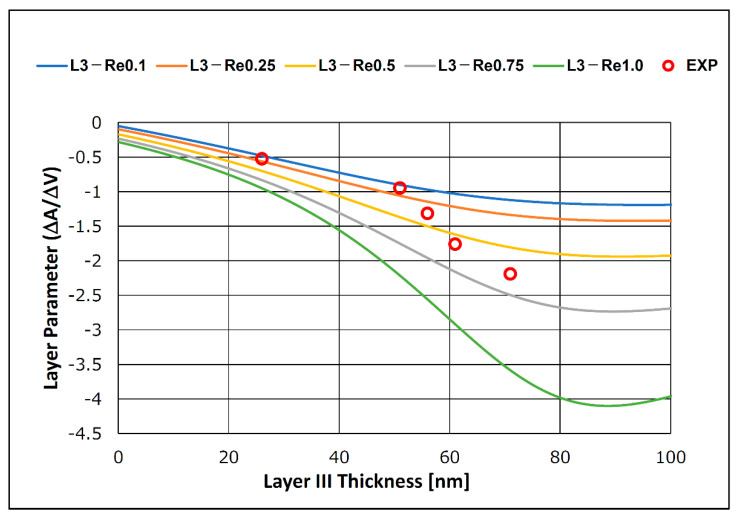
Calculated layer parameter and experimental results.

Figure 14 shows the calculated SH-SAW velocity and amplitude changes in response to a change in the thickness of the biomolecule layer. The real part of the shear modulus of elasticity was assumed to be 0.1, 0.25, 0.5, 0.75, and 1.0 MPa to compare the biomolecular layer characteristics with the measurements from the experimental results. The imaginary part of the shear modulus of elasticity was fixed at 3.0 MPa for the calculation. In the 0–40 nm biomolecular film region, the calculated results show a decrease in the SH-SAW velocity and amplitude as the biomolecular film thickness increased. The calculated results show a more considerable SH-SAW amplitude change as the SH-SAW velocity decreases and the biomolecular layer thickness increases. If the biomolecular film thickness change in the simulation could be considered as the gold nanoparticle diameter change in the experiments, this simulation result agrees with the results obtained in the experiments. Using the obtained velocity and amplitude changes from the calculation, the layer parameters for the simulation system are shown in Figure 15. The experimentally obtained layer parameters are shown in Figure 15. In the graph, the molecular length of the capture and secondary antibodies was assumed to be 15 nm, the CRP molecular size was assumed to be 11 nm, and the sum of the gold nanoparticle diameter corresponded to the biomolecule layer thickness in the simulation. The change in the thickness of the biomolecular film with a specific shear modulus of elasticity and the change in the diameter of the gold nanoparticles conjugated to the secondary antibody appear as similar changes in the layer parameter, as shown in Figure 15.

### 4.4. Considerations on the Layer Parameter in Practical Application

The results suggest that the thickness of the biomolecular film formed on the sensing region of the SH-SAW biosensor can be evaluated using the layer parameter. This property could be applied to estimate the size of the antibody molecules trapped on the sensor surface using SH-SAW biosensors. The layer parameter may also provide an index for optimizing the SH-SAW velocity and amplitude changes when selecting detection antibodies during the development phase of SH-SAW biosensors. These features suggest the usefulness of the layer parameter in SH-SAW biosensor applications.

## 5. Conclusions

In this study, we characterized SH-SAW biosensors through an antigen–antibody reaction to detect CRP using secondary antibodies conjugated with gold nanoparticles of various diameters. The layer parameter was constant even when the CRP concentration changed; further, it was affected by the shape and size of the surface-absorbed antigens and antibodies. The amplitude and velocity changes of SH-SAW with the thickness of the biomolecule layer were obtained using numerical simulations. The layer parameter calculated using simulated results demonstrated the same trends as that of the layer parameter obtained after measuring the CRP antigen–antibody reaction with various gold nanoparticle diameters. The sandwich assay protocol used in this study is a standard method, and the correlation between the layer parameters and molecular shape found in this study can be widely applied. The layer parameter property could be applied to SH-SAW biosensors for estimating the size of antibody molecules trapped within the sensor surface. Specifically, the layer parameter can distinguish the difference in shape or size between a single CRP antigen and a complex of a secondary antibody and gold nanoparticles. Moreover, the difference in the diameter of the gold nanoparticles, which are bound to the secondary antibody, can be identified. This function works in parallel with conventional specimen concentration detection, so that molecular information can be acquired simultaneously with the concentration measurement. The new function of applying the layer parameter found in this study suggests that it will enhance the usefulness of SH-SAW biosensors in POCT applications.

## Figures and Tables

**Table 1:** Experimental conditions.

Item	Experimental Variables
Diameters of gold nanoparticles	10, 15, 20, 30 nm
Operating frequency of SH-SAW biosensor	250 MHz

**Table 2:** Design parameters of the 250 MHz SH-SAW biosensor.

Item	Design Parameters
Wavelength	20 μm
Sensing length (200λ)	4000 μm

**Table 3:** Measurement process.

Step#	Reagents	Reaction Time
I	TBST	1 min
II	CRP antigens	3 min
III	TBST	1 min
IV	Secondary Ab + AuNPs	3 min
V	TBST	1 min
VI	HCl	2 min
VII	TBST	1 min

**Table 4:** Calculated regression line slopes.

Loaded Molecule	Slope of Regression Line
CRP	−0.523
2Ab + AuNPs 10 nm	−0.947
2Ab + AuNPs 15 nm	−1.315
2Ab + AuNPs 20 nm	−1.759
2Ab + AuNPs 30 nm	−2.190

## Data Availability

Not applicable.

## Supplementary Materials

The following are available online at https://www.mdpi.com/article/10.3390/s21144924/s1. For stability of antibody–gold nanoparticle complex and regeneration durability of capture antibody.
