# Supplementary material for: Evaluation of Shear Horizontal Surface Acoustic Wave Biosensors Using “Layer Parameter” Obtained from Sensor Responses during Immunoreaction
Source: Sensors (Basel). 2021 Jul 20;21(14):4924. doi: 10.3390/s21144924 (PMC8309872; doi:10.3390/s21144924)
Supplement: Supplementary file 1 [file sensors-21-04924-s001.zip › sensors-1250493-supplementary.pdf]

Supplementary Material 1

To demonstrate the stability of the gold nanoparticle-antibody complex, accelerated life tests were performed on the gold nanoparticle complexes. The secondary antibody-conjugated gold nanoparticles were stored at various temperature acceleration conditions 4°C for 7 days. The storage conditions are listed in Table S1. Assuming that the chemical reaction is accelerated by a factor of 2 for every 10°C, a sample stored at 45°C for 7 days corresponds to 120 days of storage at 4°C. At the end of the storage period, the correlation between the storage period and the degradation of the secondary antibody gold nanoparticle complex was evaluated by measuring the antigen-antibody reaction with the SH-SAW biosensor. In this evaluation, CRP antigen (1μg/mL) and secondary antibody-gold nanoparticle complex were pre-mixed and reacted with supplementary antibodies, and the sound velocity change of SH-SAW generated in the process was measured. The measurement results are shown in figure S1 and table S1. The results show that the conjugated secondary antibody-gold nanoparticle complex activity is maintained for a long time.

Table S1. Storage temperature, period and equivalent storage period

Storage Condition #	Storage Temp. [°C]	Storage Period [days]	Equiv. Period [days]
I	4	7	7
II	37	4	39
III	37	7	69
IV	45	4	69
V	45	7	120

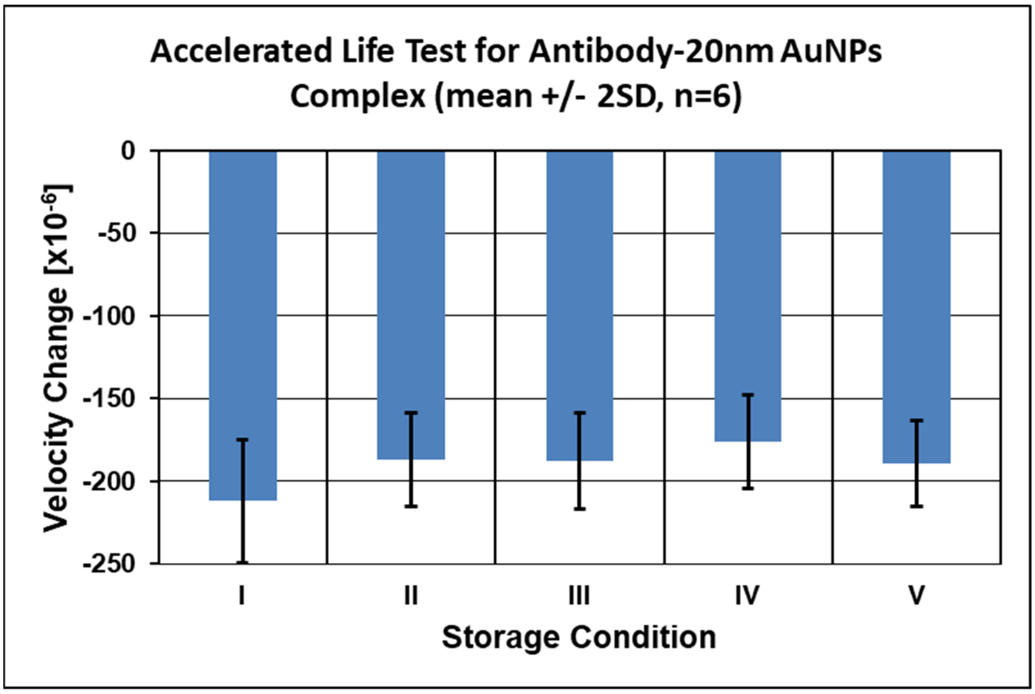

Figure S1. Accelerated life test for antibody-gold nanoparticle complex.

Supplementary Material 2

To evaluate the immune response, the event of CRP antigen adsorption on the immobilized capture antibody on the SH-SAW sensor was measured as the SH-SAW sound velocity and amplitude change. Considering the individual variability of SH-SAW sensor devices and the variability in the formation of immobilized capture antibodies, the same sensor device was used repeatedly. To use the capture antibody repeatedly, the capture antibody after the antigen-antibody reaction changes its shape and releases the adsorbed antigen when the pH value of the buffer solution changes. The pH change was achieved by loading 100 mM hydrochloric acid (HCl). From this point of view, it is desirable that the antibody used for the supplementary antibody retains its antigen-antibody activity even after repeated exposure to 100 mM HCl. Therefore, we evaluated the durability of the capture antibody to repeated HCl regeneration. A sandwich assay of CRP antigen and secondary antibodies on the SH-SAW device on which the capture antibody was performed. The assay protocol is shown in Table S2-1. The sandwich assay was repeated four times, and the change in SH-SAW velocity during antigen adsorption was recorded in each cycle. Figure S2 shows the change in SH-SAW sound velocity over time obtained from the repeated evaluation. The cycle-by-cycle decrease in the amount of SH-SAW velocity change caused by the adsorption of the CRP antigen was confirmed. The measurement results are presented in Table S2-2. The evaluation confirmed that the ability of the capture antibody to adsorb CRP antigen did not decrease even after repeated HCl regenerations.

Table S2. Regeneration Evaluation Assay Protocol.

Step#	Reagents	Reaction time
I	TBST	1 min.
II	CRP-antigens 9μg/mL	3 min.
III	TBST	1 min.
IV	Secondary Ab 120μg/mL	3 min.
V	TBST	1 min.
VI	100mM-HCl	2 min.
VII	TBST	1 min.

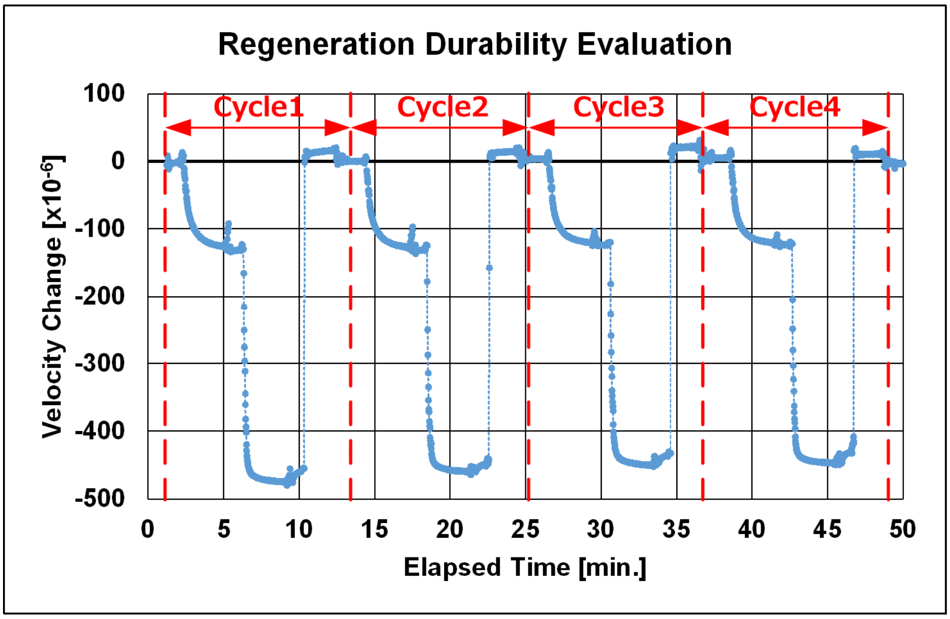

Figure S2. Recorded SH-SAW velocity change in regeneration durability test.

Table S3. Velocity change in Ag binding process for each measurement cycle.

**Cycle#**	**Velocity Change in Antigen binding [x10^{-6}]**
1	-132.7
2	-131.1
3	-127.4
4	-130.8
